# Patterns of chasmogamy and cleistogamy, a mixed-mating strategy in an endangered perennial

**DOI:** 10.1093/aobpla/plx059

**Published:** 2017-11-07

**Authors:** Stephanie M Koontz, Carl W Weekley, Sarah J Haller Crate, Eric S Menges

**Affiliations:** The Plant Ecology Program, Archbold Biological Station, FL, USA; Longleaf Program Coordinator, North Carolina Forest Service, North Carolina Department of Agriculture and Consumer Services, NC, USA

**Keywords:** Amphicarpy, chasmogamy, cleistogamy, flower dimorphism, resource availability, spatial and temporal variation

## Abstract

Cleistogamy (CL) in angiosperms historically has been understudied; however, its co-occurrence with chasmogamy (CH) across many plant species suggests a fitness advantage to maintaining this mixed-mating strategy. Maintenance of mixed-mating has been attributed to reproductive assurance, resource allocation or genetic trade-offs. Our goals were to explore patterns of CH and CL, quantify reproductive contributions measured by fruit production and determine how CL is maintained in the endangered perennial *Polygala lewtonii.* This species exhibits CH and both above-ground cleistogamy (CL-AG) and below-ground cleistogamy (CL-BG). In monthly censuses from 2008 to 2012, we documented flowering patterns by counting CH flowering stems, CL-AG fruits and CL-BG rhizomes per plant. Monitoring of buds on CH flowering stems in 2004 provided an estimate of CH fruits per plant. Plant excavations in 2005 of CL-BG rhizomes provided an estimate of CL-BG fruits per plant. Floral morphs were temporally separated with CH flowers observed from January to May and CL flowers from June to February. Overall, 17.5 % of plants flowered; most plants expressed CH first in spring months (63.4 %) and the rest initiated CL-AG in fall months. Reproductive output was dominated by CH (median 26 fruits) compared to combined CL (median 3.5 fruits). Annual reproductive effort of CL-AG was positively correlated with plant age while CH had no relation. Our research shows CH as the dominant form of reproductive effort with most individuals expressing CH and through greater reproductive contributions. CL appears limited by plant size or resources based on the positive relationship with plant age. CL dependency on resource availability is common in other species found in dry or low-quality habitats; however, CL contributions in this species are comparatively low. This raises more questions related to energy requirements of both floral morphs, how this affects the production of viable progeny and why CL persists.

## Teaser

Our research explores patterns of mixed-mating in the endemic, federally listed Polygala lewtonii. This perennial herb has open-pollinated chasmogamous flowers and self-pollinated cleistogamous flowers on both above- and below-ground stems. Data collected from 2008 - 2012 shows temporal separation of chasmogamy (January to May) and cleistogamy (June to February) with little overlap. A quarter of plants flowered all three flower morphs at least once during their lifespan while slightly less than half produced chasmogamous and above-ground cleistogamous flowers but no below-ground structures. Estimated fruit maturation was found to be seven times greater for chasmogamy compared to both forms of cleistogamy.

## Introduction

Mixed-mating in plants can provide a unique fitness advantage through the production of genetically diverse progeny while preserving locally adapted alleles. This mixed-mating was once thought to be evolutionarily unstable; however, multiple reproductive strategies occur frequently in vascular plants, with 42 % of species examined exhibiting some form of mixed reproduction (Goodwille *et al.* 2005). In a separate review, cleistogamy (CL; closed, self-pollinating flowers) was documented in 693 species across 50 families and 77 % of these also had chasmogaous, presumably outcrossed flowers (dimorphic CL; Culley and Klooster 2007). The frequency in which mixed-mating strategies have occurred suggests there is strong selection pressure for producing mixed progeny.

Strategies for mixed-mating systems can occur with the production of open-pollinated chasmogamous flowers and permanently closed self-pollinating cleistogamous flowers (Lord 1981; Schoen and Lloyd 1984; Culley and Klooster 2007), with both self-compatible and incompatible individuals (Stone 2002) or with individual flowers forming either outcrossed or selfed fruits (Schoen and Brown 1991). Chasmogamous flowers, when cross-pollinated, produce genetically diverse progeny, thus maintaining genetic diversity, while cleistogamous progeny possess only maternal information and preserve locally adapted genes (Schoen and Lloyd 1984; Waller 1984; Mitchell-Olds and Waller 1985; Schmitt *et al.* 1985; Winn and Moriuchi 2009). Chasmogamy (CH) usually relies on pollinator availability for pollen transfer, although self-pollination is also possible in some species. Chasmogamous flowers typically are energetically more expensive to produce and have lower seed set compared to cleistogamous flowers (Schemske 1978; Waller 1979; Schoen and Lloyd 1984; Mitchell-Olds and Waller 1985). CL increases a populations’ susceptibility to genetic drift and inbreeding depression if deleterious alleles cannot be purged (Lloyd 1979; Lande and Schemske 1985). These fitness trade-offs are factors in maintaining a mixed-mating strategy.

There are several hypotheses explaining natural selection leading to the maintenance of mixed-mating strategies (Goodwillie *et al.* 2005; Oakley *et al.* 2007). Reproductive assurance describes selfing as a backup mechanism when pollen is limiting or stochastic events occur (Le Corff 1993; Masuda and Yahara 1994; Culley 2002). Here, production of cleistogamous flowers is dependent on the relative success of CH and floral morphs are separated temporally or spatially to ensure progeny success (Berg and Redbo-Torstensson 1998). Another hypothesis is that allocation of resources to different floral morphs optimizes the use of available energy reserves (Schemske 1978; Schoen and Lloyd 1984). With resource allocation, production of both floral morphs is independent of each other but one or both are correlated with a resource, typically size (Waller 1980; Steets and Ashman 2004) or pollinator availability (Culley 2002). Mixed floral morphs may also be stable by a genetic balance between selfing and cross-pollination. Selfing is more effective at purging deleterious alleles (Lande and Schmske 1985; Charlesworth and Charlesworth 1987) while genetically more diverse outcrossed progeny are maintained through heterosis (Lu 2002; Oakley *et al.* 2007).

Here, we document the pattern of mixed-mating in the rare *Polygala lewtonii* (Polygalaceae), a federally endangered perennial herb (USFWS 1999; Coile and Garland 2003) found on only two ancient sand dune ridges (Mount Dora and Lake Wales Ridges) in central Florida. Its primary habitat is sandhill, dominated by long- leaf pine-wiregrass assemblages on xeric yellow sands, an ecosystem shaped by frequent fire (1–10 years; Myers 1990; Menges 1999) and seasonal fluctuations in rainfall (dry winters and wet summers) and temperature (summer temperatures > 30 °C; Menges 1999). *Polygala lewtonii* adults are killed by fire but seedlings recruit post-fire from a persistent soil seed bank (Weekley and Menges 2012). In the absence of fire, populations decline and may disappear above-ground.

*Polygala lewtonii* is one of three species within the family Polygalaceae exhibiting CL (Lord 1981; Culley and Klooster 2007). Both *P. polygama* and *P. pauciflora* exhibit amphicarpy and observational studies suggest their mixed-mating systems are maintained through resource allocation (Shaw 1901). CL in *P. lewtonii* was first briefly described by James (1957) as the species’ ability to set seed in both open and closed flowers. Small dark purple to pink chasmogamous flowers are clustered on terminal racemes. Chasmogamous flowers rely on insect pollinators and delayed selfing is rare (Weekley and Brothers 2006). Aerial cleistogamous flowers are inconspicuous, green to pale pink and solitary in the lower leaf axils. Subterranean CL occurs on rhizomes extending from the base of the plant. A recent study examining the spatial genetics of *P. lewtonii* suggested most recruitment is from cleistogamous seeds (Swift *et al.* 2016). For the remainder of this study we refer to chasmogamy as CH, above-ground cleistogamy as CL-AG and below-ground cleistogamy as CL-BG.

Understanding reproductive patterns and limitations of *P. lewtonii* can provide needed insight to its reproductive ecology and better inform conservation efforts. The goal of this study was to characterize CH and CL in this rare *Polygala*. Our objectives were to (i) describe flowering trends and frequencies of all three floral morphs (CH, CL-AG and CL-BG) since they have not been previously described for *P. lewtonii*, (ii) quantify the reproductive output of the three floral morphs and (iii) explore the selective pressures associated with maintaining a mixed-mating strategy.

As previously discussed, there are several hypotheses supporting the maintenance of mixed-mating. We predict that if CL occurs as a reproductive assurance strategy, there would be a negative correlation with chasmogamous fruit production. However, if CL is maintained by partitioning of available energy reserves (resource allocation hypothesis), we expect cleistogamous fruit production to correlate with plant size or age but to have no direct association with chasmogamous fruit production. We also explore pessimistic (initiating CL first) and optimistic strategies (initiating CH first) of initiating one floral morph over another, as first described for annual grasses (Zeide 1978; Cheplick and Quinn 1982) but also observed in perennial grass species (Campbell *et al.* 1983). These two strategies balance between producing genetically unique offspring (CH; optimistic) and producing any offspring at all (CL; pessimistic) in stochastic conditions.

## Methods

**Figure 1.**
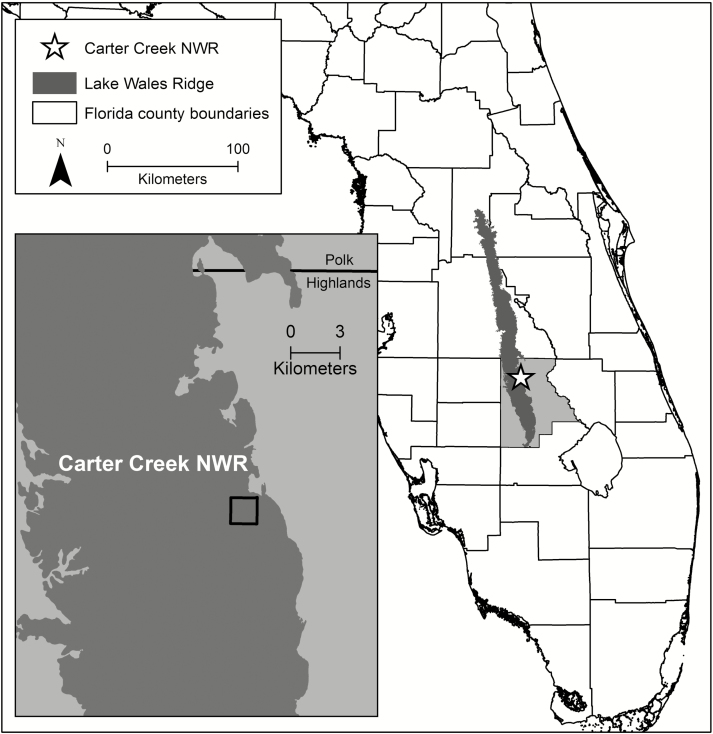
Map showing location of *Polygala lewtonii* populations at the Lake Wales Ridge National Wildlife Refuge Carter Creek along the Lake Wales Ridge in Highlands County, Florida.

We followed individuals of *P. lewtonii* at the Lake Wales Ridge National Wildlife Refuge (LWRNWR) Carter Creek located in south-central Florida (Fig. 1). This refuge is predominantly xeric sandhill and is managed with prescribed fire, most recently occurring in 2001, 2007 and 2009; the 2009 prescribed fire did not affect our study area. Data on individual plants and counts of CL-AG mature fruits were collected from 2008 to 2012. Specific counts of mature fruits on CH stems and CL-BG rhizomes were collected in 2004 and 2005, respectively, on separate individuals.

### Data collection

Plants were followed monthly from germination to senescence from March 2008 through December 2012 (excluding July 2008) for a total of 57 censuses. Indivi dual plants were marked with pin flags and unique numbered aluminium tags.

Seedling survival in this species is low with >50 % of a cohort dying within their first year (Weekley and Menges 2012) making it difficult to maintain an adequate sample size. To combat this problem, we added new recruits during monthly censuses as needed throughout the study. Therefore, plants followed in this study are not from the same cohort but were all followed from germination to senescence or until the study’s termination.

At each monthly census, we recorded survival, counted vegetative stems and quantified reproductive activity of the three flower morphs. Vegetative stems were counted on all individuals until CH flowering stems formed, after which only active CH flowering stems were counted. The number of mature CH fruits were not counted but estimated using a separate data set (see below). CL-AG mature fruits were counted for each plant at each monthly census. CL-BG rhizomes were counted by carefully excavating around the base of multi-stemmed (>1 vegetative stem) plants and counting any extending rhizomes. The presence of rhizomes suggested below-ground reproductive activity but entire rhizomes were not excavated to count flowers and fruits due to the destructive nature of such excavations. Mean fruit production per rhizome of CL-BG was estimated using a separate data set (see below).

### Estimating fruit production

To compare reproductive effort of these three floral morphs, counts of mature fruits per plant were needed. During 2008–2012 monthly census, the total number of mature CL-AG fruits were counted but not for CH or CL-BG. To obtain median fruit production per plant for CH and CL-BG, we used two separate data sets collected from the same site in 2004–2005.

In March 2004, 20 plants were randomly selected to monitor CH flower and fruit development as part of another study (Weekley and Brothers 2006). On each plant, three random bud-bearing CH stems were marked using colour-coded thread for identification and the number of buds per stem counted. Plants were monitored for 59 days as buds developed. Fruits were mature if abscission occurred with a slight touch or within 22 days after initiation. Using these data, we calculated a median number of mature fruits produced per CH stem.

In April 2005, 40 plants of various sizes were selected to excavate for below-ground rhizomes. All plants were carefully excavated to reveal the full length of all rhizomes and to quantify all reproductive structures (buds, flower, fruits and capsules). Not all rhizomes had below-ground structures and we assumed all reproductive structures matured. We used these data to quantify the median number of mature fruits per rhizome.

### Statistical analysis

We used descriptive statistics to compare median age of reproductive maturity and the frequency of each floral expression, and a chi-squared test to determine the probability of initiating a floral morph.

#### Estimating reproductive output.

CL-AG flowers are small, inconspicuous and mature rapidly; therefore, counts may underestimate true CL-AG production. CH flowering stems and CL-BG rhizomes often persist from month to month meaning some stems may have been counted twice, overestimating reproductive output for these two morphs. To reduce over estimates, the peak number of CH stems and CL-BG rhizomes observed during each reproductively active season was used as the maximum number of reproductive stems/rhizomes for that individual for that season. We acknowledge reproductive output varies by individual and year. Our estimates of CH and CL-BG fruit production are based on data collected from different plants in a different year however, more exact counts were not feasible due to time constraints and the destructive nature of excavating CL-BG rhizomes.

We calculated the number of mature fruits produced per plant based on the annual peak number of CH stems observed during monthly censuses. The same was done for CL-BG using the annual peak number of rhizomes observed. This gave us an estimate of peak annual fruit production for CH and CL-BG per plant to compare with CL-AG.

#### Hypotheses maintaining mixed-mating.

Linear mixed models examined the relationship between peak annual CH and CL-AG fruit production and determined how fruit production for both floral morphs varied with plant age. In this study, we used plant age as a proxy for plant size based on marked individuals in permanent plots. A separate demographic data set showed 14 years of size measurements taken annually in March determined plant age and size are correlated in *P. lewtonii* (*N* = 1287, Pearson’s *r* = 0.32, *P* < 0.0001; Weekley and Menges 2012). Both models included individual as a random effect to account for repeated measures on individual plants. Regression analyses were run on significant fixed factors to determine the direction and strength of any significant relationships identified in mixed models. All fruit counts were natural log transformed to fit normality assumptions. All analyses were done in SPSS version 22.0 (IBM Corp. 2013).

## Results

Our study captured data on 234 seedlings of *P. lewtonii* from germination to senescence with 17.5 % (*N* = 41) surviving to reproductive maturity. Reproductive maturity was defined as flowering CH or CL-AG at least once; some plants expressed only a single floral morph. Seedling recruitment occurred year-round with peak germination in spring months; therefore, our sample size was biased with most seedlings germinating from February through April also surviving to reproduction (Table 1).

**Table 1.** Recruitment months of 234 *Polygala lewtonii* seedlings, their survival to reproduction and percent of plants initiating CH first. Otherwise, plants initiated above-ground CL first. No plants produced below-ground CL first.

Month	# recruits	% survived to reproduction	% flower CH first
January	4	0 %	–
February	50	14.0 %	71.4 %
March	69	29.0 %	65.0 %
April	29	13.8 %	25.0 %
May	21	19.0 %	100 %
June	0	–	–
July	0	–	–
August	7	29.6 %	50.0 %
September	13	0 %	–
October	6	16.7 %	100 %
November	0	–	–
December	35	8.6 %	33.3 %
Total	234	17.5 %	63.4 %

### Flowering trends and frequencies

Among observed plants, 24.4 % produced only CH flowers, 9.7 % produced only CL-AG flowers, 41.5 % flowered both CH and CL-AG and 24.4 % flowered all three floral morphs (Table 2). Median age for initiating CH flowers was 23 months compared to 19 months for CL-AG and 29.5 months for CL-BG. There was a marginally significant difference in the probability of initiating CH or CL-AG first (χ^2^ = 2.951, df = 1, *P* = 0.086); almost two-thirds of plants initiated CH first (63.4 % vs. 36.6 % CL-AG first; Table 2). No plants in this study initiated CL-BG first.

**Table 2.** Expression of CH, above-ground and below-ground cleistogamy (CL) in 41 reproductive individuals of *Polygala lewtonii* followed from germination through senescence. Frequency shows the percentage of plants that expressed either a single or multiple flower morphs (*N*_1_ = number of plants producing each combination of floral morphs). Initial floral morph shows the percentage of plants that initiated CH or CL first (*N*_2_ = number of plants that first initiated a specific floral morph first). Median age and range in months for when each floral morph was first observed regardless of initial floral morph (*N*_3_ = number of plants that expressed each floral morph).

Floral morph	Frequency (*N*_1_)	Initial floral morph (*N*_2_)	Median age in months; range (*N*_3_)
Chasmogamy (CH)	24.4 % (10)	63.4 % (26)	23; 7–45 (37)
Above-ground cleistogamy (CL-AG)	9.7 % (4)	36.6 % (15)	19; 11–41 (31)
Below-ground cleistogamy (CL-BG)	0 % (0)	0 % (0)	29.5; 16–44 (10)
CH and CL-AG	41.5 % (17)	–	–
CH, CL-AG and CL-BG	24.4 % (10)	–	–

### Estimating reproductive output

Mature CH fruits per stem ranged from 0 to 24 with a median of 13 fruits (mean = 12 ± 5 SD). Using this median, we estimated CH fruit production to be a median of 26 fruits per plant ranging from 13 to 182 fruits (mean = 45.6 ± 40.9 SD).

Fifteen of the 40 plants had 1–10 rhizomes (median = 3) with a median of 1 reproductive structure per stem (range 0–14; mean = 2.8 ± 3.3 SD). Assuming all below-ground reproductive structures matured, we calculated a median of 1.5 fruits per plant with a range of 1–6 fruits (mean = 2.3 ± 1.7 SD).

Plants with CL-AG produced a range of 1–42 mature fruits with a median of 2 per plant (mean 4.4 ± 7.7 SD) compared to 1.5 CL-BG fruits and 26 CH fruits. Even the combined output of CL (3.5 mature fruits) was less than CH reproductive effort.

### Hypotheses maintaining mixed-mating

**Figure 2.**
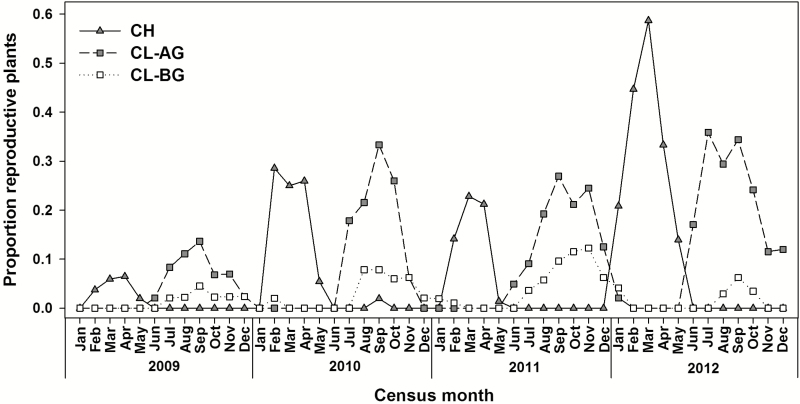
Monthly CH and CL flowering/fruiting patterns of *Polygala lewtonii* from 2009 to 2012. Plants were monitored monthly and recorded as reproductively active with chasmogamously (CH) flowering stems, above-ground cleistogamous (CL-AG) fruits or rhizomes on which below-ground cleistogamous (CL-BG) fruits are found.

CH and CL flower and fruit production showed temporal separation with little overlap (Fig. 2), making *P. lewtonii* reproductively active all year. CH flowering stems were found from January to May. CL production followed shortly after with CL-AG fruits found from June to January and CL-BG rhizomes from July to February.

**Figure 3.**
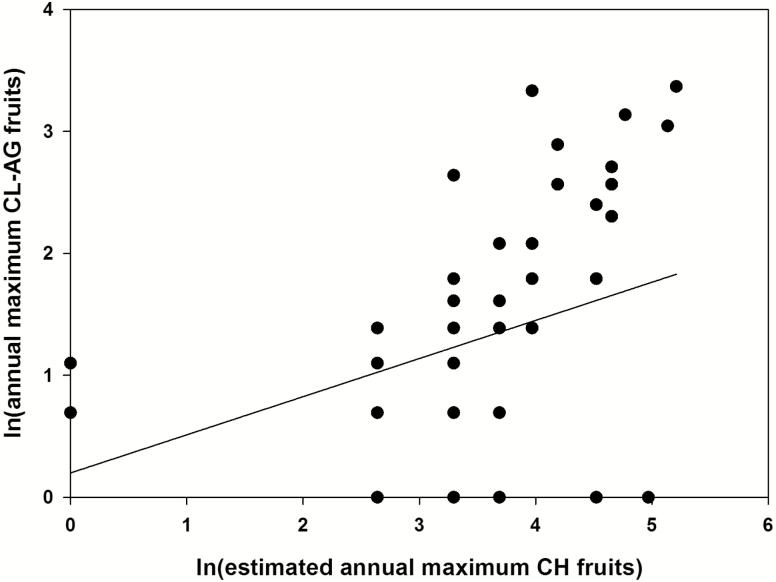
Linear regression model of maximum annual mature fruit production for chasmogamous (CH) and above-ground cleistogamous (CL-AG) flower morphs of *Polygala lewtonii* (*N* = 63, some points overlap). The regression shows a significant positive relationship in mature fruit production between the two flower morphs (*r*^2^ = 0.23, *P* < 0.001).

Linear mixed models showed there was a marginally significant relationship between CH and CL-AG fruit production (*F*_1, 62.3_ = 3.95, *P* = 0.051) with significant variation in production of both floral morphs between individuals (Wald *Z* = 3.024, *P* = 0.002). Regression analysis showed a weak positive but significant relationship between CH and CL-AG fruit production (*r*^2^ = 0.23, *F*_1, 81_ = 23.968, *P* < 0.001; Fig. 3).

**Figure 4.**
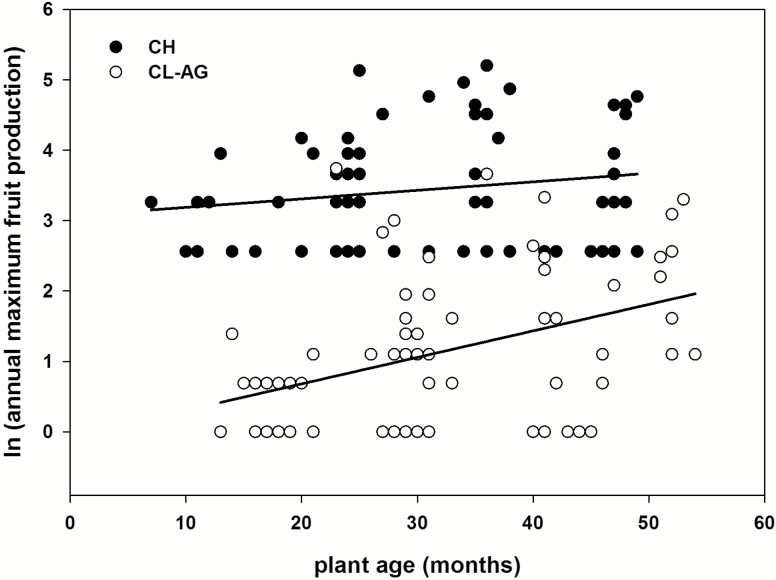
Linear regression model of plant age and maximum annual fruit production of chasmogamous (CH) and above-ground cleistogamous (CL-AG) flower morphs. There was a significant relationship of CL-AG fruit production (*r*^2^ = 0.16, *P* < 0.001) with age and a marginal relationship of CH fruit production with age (*r*^2^ = 0.04, *P* = 0.050).

Peak seasonal fruit production varied significantly by floral morph (*F*_1, 117.7_ = 107.52, *P* < 0.001) and the interaction of morph with age (*F*_1, 115.9_ = 6.09, *P* = 0.015) but not age alone (*F*_1, 135.0_ = 0.81, *P* = 0.368). Fruit production also varied by individual (Wald *Z* = 3.19, *P* = 0.001). Breaking down the interaction by floral morph shows no significant effect of age on CH fruit production (*F*_1, 72.2_ = 2.24, *P* = 0.139) but a significant effect on CL-AG fruit production (*F*_1, 67.7_ = 11.41, *P* = 0.001). Regression analyses showed a weak but significant positive relationship with plant age and CL-AG fruit production (*r*^2^ = 0.16, *F*_1, 66.3_ = 13.9, *P* < 0.001) and no relationship with CH fruit production (*r*^2^ = 0.04, *F*_1, 74_ = 3.98, *P* = 0.050; Fig. 4).

## Discussion

Mixed-mating systems have evolved independently many times with several hypotheses addressing how these systems are maintained. Our results show a marginal probability for initiating chasmogamous flowers before above-ground cleistogamous flowers, greater chasmogamous reproductive effort, temporal separation between CH and CL flowering periods and a positive relationship between plant age and AG-CL fruit production. Temporal separation of floral morphs suggests CH and CL are favoured under different seasonal conditions and have different resource limitations (Schoen and Lloyd 1984). We found no evidence for CL acting as reproductive assurance for failed chasmogamous production. Instead, CL was positively correlated with CH indicating no trade-off in resource allocation to either floral morph. Finally, chasmogamous fruit production was estimated to be over seven times that of cleistogamous fruits (above- and below-ground combined).

CL has been shown to occur prior to CH in response to resource stress (Cheplick and Quinn 1982; Campbell *et al.* 1983) or after adequate resources and growth have been obtained in both annual and perennial grasses (Zeide 1978; Cheplick and Quinn 1982). Our data show CL was positively related to plant age and occurred prior to CH in one-third of plants, with some individuals never producing chasmogamous flowers. Thus, CL in *P. lewtonii* appears to be resource dependent while CH may be restricted by another resource such as pollinator availability (Culley 2002).

In many species, CL is expressed as a response to resource availability. Plant size has been shown to be a limiting factor for reproductive effort (Jasieniuk and Lechowicz 1987; Diaz and MacNair 1998; Munguía-Rosas *et al.* 2015) with some studies demonstrating that manipulations of above-ground vegetation can significantly reduce CL alone (Diaz and MacNair 1998) or both floral morphs (Munguía-Rosas *et al.* 2015). Resource limitations that negatively impact CL may be induced by environmental stresses such as soil moisture or soil fertility gradients (Schoen and Lloyd 1984; Bell and Quinn 1987; Albert *et al.* 2011). However, in a review by Campbell *et al.* (1983), two-thirds of perennial non-ruderal grass species expressed CL in response to stochastic environmental conditions.

In *P. lewtonii*, chasmogamous fruit production was over seven times greater than that of above- and below-ground CL combined, a pattern that does not fit with other studies of CL. Most species with mixed-mating have higher cleistogamous seed production or produce larger seeds, and several studies found that cleistogamous progeny out-performed chasmogamous progeny across several developmental stages (Schemske 1978; Clay 1983; Waller 1984; Sun 1999; Culley 2002; Winn and Moriuchi 2009). Cleistogamous seeds in *P. lewtonii* are larger than chasmogamous seeds (C. W. Weekley, Archbold Biological Station, pers. comm.), although successful seed set and progeny fitness of both floral morphs were not explored in this study. Limited CL suggests poor environmental conditions outside the ideal growing season but under more favourable conditions all modes of reproduction are more successful (Jones *et al.* 2015). High chasmogamous reproductive effort was observed in the current study and high chasmogamous fruit maturation was found by Weekley and Brothers (2006) even with low insect visitation. However, autogamy is rare in *P. lewtonii*, based on low chasmogamous fruit maturation in a pollinator exclusion experiment (Weekley and Brothers 2006). At this time, we have no explanation for the discrepancy in observed chasmogamous and cleistogamous fruit maturation.

The mixed-mating system of *P. lewtonii* is separated temporally, with selfing by CL positively associated with resource availability. It is still peculiar that chasmogamous reproductive effort exceeds cleistogamous efforts the reverse of what is seen in most other species. A recent population genetic study found that most individuals surviving to adulthood are progeny from cleistogamous seeds (Swift *et al.* 2016), suggesting a higher fitness for cleistogamous progeny. This also raises the question of why so much effort is being put into CH if few outcrossed progeny are represented in the next generation of reproductive individuals. More research is needed to understand the apparent failure of CH to produce viable offspring, even as more effort is allocated to producing these seeds. Additionally, we need a better understanding of how environmental gradients affect resource allocation for reproduction. These two key topics would add valuable knowledge to the reproductive biology of *P. lewtonii* and aid in highlighting conservation concerns (such as limited pollinator availability) for this species.

## Conclusions

The occurrence of mixed-mating should be evolutionarily unstable, but has been documented in many vascular plants (Goodwillie *et al.* 2005; Culley and Klooster 2007) including in the rare *P. lewtonii.* We found a pattern of strong seasonal separation between CH and CL flower production, a positive correlation between mature cleistogamous fruit production and plant age, and initiation of CH in two-thirds of plants prior to CL. These patterns have been observed in other species with mixed-mating in low-quality habitats with variable rainfall (Campbell *et al.* 1983; Jones *et al.* 2015) and are linked to resource requirements. Temporal separation in floral morphs allows *P. lewtonii* to be reproductively active year-round in a stochastic environment dominated by dynamic rainfall events and fluctuating temperatures, but raises concerns about the amount of failed effort contributed to outcrossed progeny.

## Sources of Funding

The Florida Division of Plant Industry and the National Science Foundation. No grant numbers are provided from the Division of Plant Industry since these were annual grants and the multiple numbers would take lots of space. They can be provided if needed. The award number for the grant from NSF is provided but is not highlights.

## Contributions by the Authors

S.M.K. completed final analyses for publication, wrote the manuscript and completed all edits. C.W.W. designed and implemented the initial project, collected data for most years and did preliminary analyses. S.J.H.C. collected data in the final years of the project and did many preliminary analyses. E.S.M. assisted with writing and editing the manuscript for publication.

## Conflicts of Interest

None declared.
